# Multi-Scale Spatio-Temporal Attention Networks for Network-Scale Traffic Learning and Forecasting

**DOI:** 10.3390/s24175543

**Published:** 2024-08-27

**Authors:** Cong Wu, Hui Ding, Zhongwang Fu, Ning Sun

**Affiliations:** 1 Engineering Research Center of Wideband Wireless Communication Technology, Ministry of Education, Nanjing University of Posts and Telecommunications, Nanjing 210003, China; wucong@njupt.edu.cn (C.W.); farprt@foxmail.com (Z.F.); 2 State Key Laboratory of Air Traffic Management System, Nanjing 210014, China; dhlshy2006@163.com

**Keywords:** traffic forecasting, spatio-temporal networks, graph convolutional networks, multi-dimensional lstm

## Abstract

Accurate and timely forecasting of traffic on local road networks is crucial for deploying effective dynamic traffic control, advanced route planning, and navigation services. This task is particularly challenging due to complex spatio-temporal dependencies arising from non-Euclidean spatial relations in road networks and non-linear temporal dynamics influenced by changing road conditions. This paper introduces the spatio-temporal network embedding (STNE) model, a novel deep learning framework tailored for learning and forecasting graph-structured traffic data over extended input sequences. Unlike traditional convolutional neural networks (CNNs), the model employs graph convolutional networks (GCNs) to capture the spatial characteristics of local road network topologies. Moreover, the segmentation of very long input traffic data into multiple sub-sequences, based on significant temporal properties such as closeness, periodicity, and trend, is performed. Multi-dimensional long short-term memory neural networks (MDLSTM) are utilized to flexibly access multi-dimensional context. Experimental results demonstrate that the STNE model surpasses state-of-the-art traffic forecasting benchmarks on two large-scale real-world traffic datasets.

## 1. Introduction

Traffic forecasting poses significant challenges within intelligent transportation systems (ITS) due to the dynamic and complex nature of traffic flow. Accurate prediction of road network conditions is crucial for deploying effective dynamic traffic control, advanced route planning, and navigation services, which mitigate congestion and enhance transportation mobility.

The objective of traffic forecasting is to anticipate future key indicators (such as speed, volume, and density) of local traffic networks based on historical traffic data and physical road network characteristics. This task is particularly demanding due to intricate spatio-temporal dependencies arising from road network topology and non-linear temporal dynamics [1]. Unlike sequential image data, traffic data naturally forms graph-structured sequences, where sensor networks exhibit non-Euclidean and directional spatial relationships. Additionally, traffic sequences exhibit strong temporal correlations between adjacent timestamps and recurring patterns over long historical periods (e.g., daily and weekly trends), posing challenges for conventional modeling approaches.

The study of traffic forecasting spans decades, typically categorized into classic statistical models and machine learning techniques. In time series analysis, methods like auto-regressive integrated moving average (ARIMA) [2,3] and Kalman filtering [4] have been traditional choices. However, these statistical models heavily rely on stationary assumptions [5], which are frequently violated by traffic data, limiting their effectiveness with high-dimensional and dynamic traffic flow data.

Recent advances in machine learning, including support vector regression (SVM) [6], Bayesian networks [7], and neural network (NN) models [8], have demonstrated promising capabilities in handling high-dimensional data and capturing complex non-linear relationships within traffic patterns. Yet, the full potential of machine learning in traffic forecasting has only been fully realized with the advent of deep learning models.

Deep learning models such as deep belief networks (DBN), stacked autoencoders [9,10], and recurrent neural networks (RNNs) [11]—including variants like long short-term memory (LSTM) and gated recurrent unit (GRU)—have excelled in learning effective features and improving prediction accuracy without the need for hand-engineered feature selection [12]. However, conventional RNN models are often constrained by practical limitations, such as their ability to handle traffic periodicities in lengthy historical sequences limited to 250–500 time steps [13,14].

While hybrid models that combine convolutional neural networks (CNNs) with recurrent neural networks (RNNs) have been explored to exploit spatial and temporal regularities [15,16,17,18], these approaches face significant challenges when extended to very long-range temporal dependencies. Standard CNNs are primarily designed to process data in regular grid structures within Euclidean space, which can result in inefficiencies when applied to complex, irregular traffic networks [19,20]. Moreover, despite advances in RNN variants such as long short-term memory (LSTM) and gated recurrent unit (GRU), these networks often struggle with issues like vanishing gradients and limited memory capacity, which constrain their ability to effectively model long-term dependencies. Recent research has sought to overcome these limitations by incorporating graph convolution techniques, inspired by graph convolutional networks (GCNs) [21,22,23,24]. While these methods have demonstrated improved capabilities in handling the irregular spatial structure of traffic data, they often fall short in maintaining predictive accuracy over extended temporal sequences. Multi-dimensional RNNs [25,26] offer some progress in modeling complex spatio-temporal dynamics, yet they continue to encounter difficulties with the computational and architectural complexity required to fully capture the intricate, long-term dependencies characteristic of traffic flow data over prolonged periods.

Recent research in traffic flow prediction has made significant strides by incorporating advanced machine learning and deep learning techniques, particularly for short-term prediction and the integration of spatio-temporal data. Ma et al. proposed a novel approach that combines time series analysis with LSTM and BiLSTM networks, demonstrating notable improvements in short-term traffic flow prediction for urban road sections [27]. Wang et al. introduced a hybrid deep learning model that integrates 1D convolutional neural networks (CNNs), LSTM, and attention mechanisms, resulting in enhanced predictive accuracy [28]. Shu et al. developed an improved gated recurrent unit (GRU) neural network model tailored for short-term traffic flow prediction, showing superior performance compared to traditional methods [29]. Berlotti et al. presented a machine-learning approach specifically designed for dynamic traffic environments, highlighting its potential in traffic flow prediction [30]. Rossi et al. proposed a measurement-independent technique for urban road traffic noise prediction by coupling different noise models with a multilinear regression model [31]. Huang et al. developed the MD-GCN, a multi-scale temporal dual graph convolutional network, to effectively capture the complex spatio-temporal dynamics of traffic flow [32]. Ji et al. introduced a spatio-temporal self-supervised learning method for traffic flow prediction, underscoring the model’s capability to learn from large volumes of unlabeled data [33]. Li et al. employed coordinated attention mechanisms within spatio-temporal models, further improving traffic flow prediction accuracy [34]. Cui et al. advanced the field with the Mfdgcn, a multi-stage spatio-temporal fusion diffusion GCN, enhancing the integration of spatio-temporal data for more precise predictions [35]. Li et al. proposed SASTGCN, a self-adaptive spatio-temporal GCN that dynamically adjusts its architecture in response to varying traffic conditions [36]. Finally, Ma et al. introduced the dynamic spatio-temporal graph fusion convolutional network, which emphasizes the dynamic fusion of spatio-temporal features to enhance urban traffic prediction [37]. Collectively, these studies underscore the rapid development of sophisticated models that integrate both temporal and spatial data to tackle the complexities of traffic flow prediction.

Despite significant progress in traffic forecasting using deep learning models, existing approaches continue to face two critical challenges: (1) effectively capturing the complex spatio-temporal dependencies inherent in traffic data, particularly within graph-structured road networks, and (2) efficiently processing very long sequences of traffic data to account for multi-scale temporal characteristics. Traditional CNNs and RNNs have demonstrated potential, but they are generally limited to regular grid structures or fall short in managing extended time series data. Although hybrid models that combine CNNs with RNNs or attention mechanisms have been proposed, these models often struggle with non-Euclidean spatial relationships and inadequately capture long-range temporal dependencies.

Recent research has focused largely on short-term traffic flow prediction, leveraging variations of LSTM networks, GRUs, and hybrid deep learning architectures. However, these approaches are often insufficient for large-scale, complex road networks where spatial and temporal dynamics are particularly intricate. Additionally, there is a scarcity of models specifically designed to learn from very long sequences of graph-structured time series data, a capability that is essential for accurate long-term traffic forecasting.

This paper seeks to address these gaps by introducing a novel deep learning framework, the spatio-temporal network embedding (STNE) model, specifically designed to learn and forecast from graph-structured traffic data over extended input sequences. The objectives of this research are as follows:

1. To develop a model that integrates graph convolutional networks (GCNs) with multi-dimensional long short-term memory (MDLSTM) networks for concurrently capturing both spatial and temporal dependencies in very long sequences of graph-structured traffic data.
2. To propose and validate the use of multi-scale temporal convolutional layers within the STNE framework, allowing the model to effectively process and learn from extended input sequences that exhibit diverse temporal properties, such as closeness, periodicity, and trends.
3. To empirically evaluate the performance of the STNE model on large-scale real-world traffic datasets, demonstrating its superiority over existing state-of-the-art traffic forecasting methods, particularly in terms of accuracy and robustness across varying network scales.
4. To establish the versatility of the STNE model by demonstrating its applicability to other domains that involve very long sequences of graph-structured time series data, such as weather forecasting and car-hailing demand–supply analysis.

The remainder of this paper is organized as follows: Section 2 outlines the proposed framework, Section 3 presents experimental results on two real-world datasets, Section 4 discusses the findings, and Section 5 concludes the paper.

## 2. Materials and Methods

### 2.1. Traffic Forecasting Problem

The goal of traffic forecasting is to utilize historical traffic flow data, such as speed and volume, to predict future traffic conditions within a specific road network composed of *N*-correlated sensors. The relationships among sensor stations are characterized by modeling as an undirected and connected graph *G* = (*V*, *E*, *A*), where *V* denotes the set of nodes (i.e., sensor stations), *E* represents the set of edges, and *A* is the adjacency matrix representing the connectedness of those nodes in the network. Denote the traffic flow measured on *G* at a given time t is represented by a graph signal *X_t_*, where *D* is the number of features of each node (e.g., speed, volume). The spatio-temporal traffic forecasting problem aims to predict the most likely future p time step of the graph signal given the historical graph signals:

(1) $${\hat{X}}_{t+p}=argmax\;P[X_{t+p}|X_{t+p},\cdots ,X_{t},G(V,E,A)]$$

### 2.2. Model Architecture

**Figure 1:**
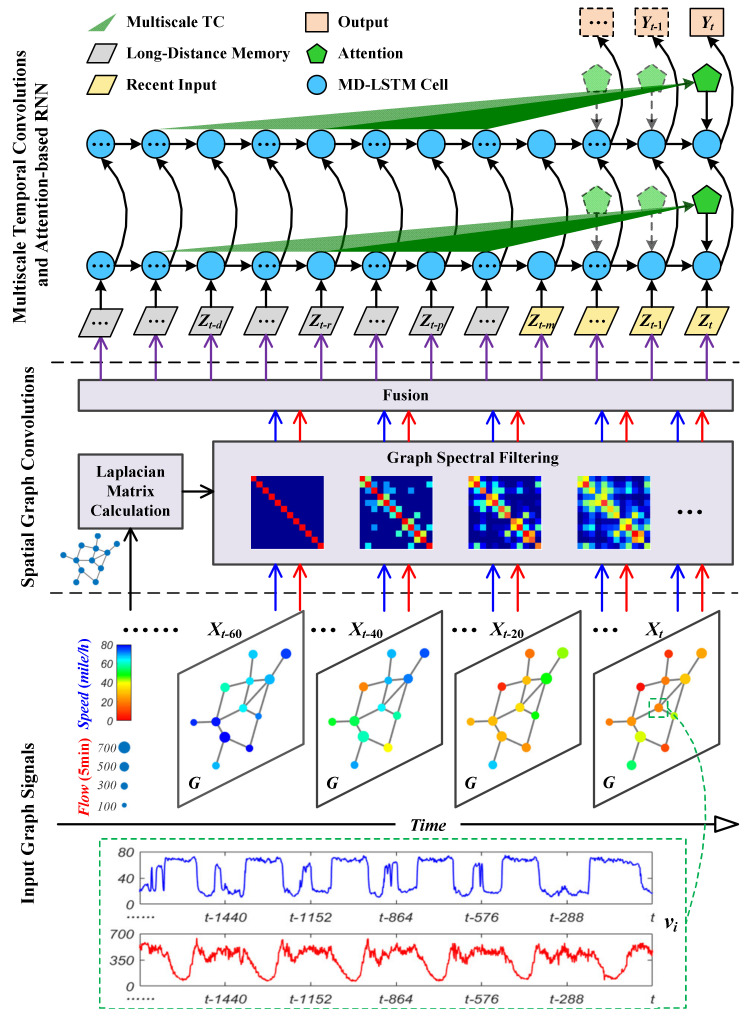
Architecture of multi-scale spatio-temporal attention networks model.

In this section, Figure 1 illustrates the overall architecture of the multi-scale spatio-temporal attention networks (MSTAN) model designed for network-scale traffic forecasting. The MSTAN integrates a graph convolutional (GC) layer, multi-scale temporal convolutions (TC), and attention-based multi-dimensional LSTM (MD-LSTM) cells.

- GC Layer: The GC layer is fundamental for capturing the spatial dependencies within traffic networks. Traffic data, collected from sensors dispersed across a road network, can be naturally represented as a graph where nodes correspond to sensors and edges represent the connections (e.g., roads) between them. The GC layer operates on this graph structure to extract spatial features, enabling the model to aggregate information from neighboring nodes and learn representations that capture the spatial relationships between various locations in the traffic network.
- Multi-Scale TC: To capture temporal dynamics over varying time scales, the MSTAN model employs multi-scale TC. TC are effective for modeling sequential data, as they apply a sliding window of filters across the time axis, allowing the model to learn temporal patterns. The multi-scale nature of TC ensures that the model uses multiple filters with different kernel sizes, enabling it to attend to temporal features at various resolutions—from short-term fluctuations to long-term trends—thereby effectively capturing the diverse temporal characteristics inherent in traffic data.
- MD-LSTM: To enhance the model’s ability to manage complex temporal dependencies, particularly over long sequences, the MSTAN integrates an attention-based MD-LSTM. LSTMs, a type of RNN, are well-suited for sequence data, capable of learning long-term dependencies through a gating mechanism that controls the flow of information. The multi-dimensional aspect allows the LSTM to process data across multiple dimensions (e.g., spatial and temporal), facilitating the capture of interactions between these dimensions. Additionally, an attention mechanism is incorporated to further enhance the model’s ability to focus on the most relevant parts of the input sequence. This mechanism enables the model to weigh different parts of the sequence according to their relevance to the prediction task at hand.

The MSTAN model effectively combines these advanced techniques to capture both spatial and temporal dependencies in traffic data. The GC layer addresses spatial correlations by leveraging the graph structure of the traffic network. Multi-scale TC capture temporal dependencies across multiple time scales, while the attention-based MD-LSTM further refines the model’s capacity to focus on the most relevant temporal features, especially over extended input sequences. This integration of methods allows the MSTAN model to achieve state-of-the-art performance in network-scale traffic forecasting. Detailed model specifications are provided in subsequent sections.

### 2.3. Spatial Attention with Graph Convolutions

- Laplacian Matrix Calculation.

In this paper, the spatial dependency on graphs is modeled using graph convolutions, which explicitly captures the topological structure of the traffic network. In graph theory, the Laplacian matrix of a graph is defined as *L* = *D* − *A*, where *A* is the adjacent matrix and *D* is the diagonal matrix with *d_ii_* = ∑*_j_ a_ij_*. The normalized version *L* = *I_n_* − *D*^−1/2^*AD*^−1/2^ is chosen, where *I_n_* is the identity matrix.

- Graph Spectral Filtering.

As *L* is a real symmetric positive semidefinite matrix, the eigen decomposition of *L* = *UΛU^T^* is diagonalized by the Fourier basis *U* and the diagonal matrix of eigenvalues. In the graph Fourier transform domain, the convolution operator on graph *G is defined such that:

x*G y = *U*((*U^T^x*)⊙(*U^T^y*)) (2)

where ⊙ is the element-wise Hadamard product. Further, it follows that a signal *x* is filtered by *g_θ_*(*L*) as:

*y* = *g_θ_*(*L*)*x* = *g_θ_*(*UΛU^T^*)*x* = *Ug_θ_*(*Λ*)*U^T^x* (3)

Therefore, *g_θ_*(*Λ*) can be regarded as the graph convolution in previous studies.

- Chebyshev Polynomials Approximation.

In order to reduce the number of parameters and localize the filter, *g*_θ_(*Λ*) can be parametrized as a truncated expansion of Chebyshev polynomials *Λ^k^* and corresponding coefficients *θ^k^* as:

(4) $$g_{\theta }(\Lambda )\approx \sum_{k=0}^{K-1}\theta_{k}\Lambda^{k}$$

where *K* is the kernel size of graph convolutions. The graph filtering operation can be written as:

(5) $$y\approx U\left(\sum_{k=0}^{K-1}\theta_{k}\Lambda^{k}\right)U^{T}x=\sum_{k=0}^{K-1}\theta_{k}L^{k}x$$

- Graph Convolutional Layer.

In this paper, the graph signal of traffic flow is denoted as *X*_G_ = [*x*_0_, *x*_1_, , *x_D-1_*], where *D* is the number of features of each node (e.g., speed, volume). *D* graph spectral filters with size *K* are adopted for graph convolutions and fusion operation as shown in Equation (6):

(6) $$\begin{array}{c} Z=F\left(\sum_{k=0}^{K-1}\theta_{k_{0}}L^{k}x_{0},\sum_{k=0}^{K-1}\theta_{k_{1}}L^{k}x_{1},\cdots ,\sum_{k=0}^{K-1}\theta_{k_{\left(D-1\right)}}L^{k}x_{\left(D-1\right)},\right)\\ =F\left(\Theta_{0}*Gx_{0},\Theta_{1}*Gx_{1},\cdots ,\Theta_{\left(D-1\right)}*Gx_{\left(D-1\right)},\right)\;\;\;\;\;\;\;\;\;\;\;\;\;\;\; \end{array}$$

where *F* is the fusion function, such as maximum and arithmetic mean rule, and *Z* is the final output of spatial features extracting with *D* graph convolutional kernels. Hence, the road network graph convolutional layer is denoted as **GC**:

*Z =***GC** (*X*, *L*, *Θ*) (7)

where *Θ* is the kernel tensor, *L* is the normalized Laplacian matrix of traffic network. In the model, the graph convolutions above are employed directly on graph structured data to extract highly meaningful spatial features and pay more attention on local road network topology.

### 2.4. Multi-Scale Temporal Convolutions

In traffic flow forecasting studies, researchers have proven that traffic time series are influenced by recent data as well as several key temporal properties in very long history, such as daily periodicity and workdays/weekends. However, it is very challenging to tackle very long input sequences when using canonical RNN or other existing models. For example, traffic flow during rush hours may be similar on consecutive workdays, and the traffic flow patterns of workdays and holidays are quite different.

- Temporal Convolution Architecture.

**Figure 2:**
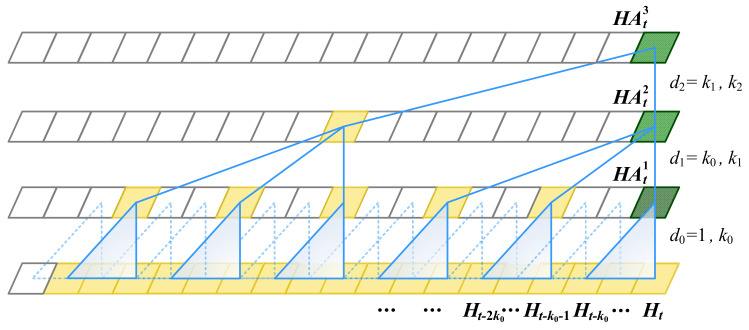
Illustration about sequences summarization.

Based on these insights, longer effective history memory was built using temporal convolution architecture, which allows the model to look very far into the past to make a prediction. In the model, the long input sequence is processed by a 1D fully convolutional network and causal convolution with no leakage from the future into the past. According to each key temporal property, multi-scale dilated convolutions are constructed for a sufficiently large receptive field and fewer parameters, as shown in Figure 2.

- Standard LSTM Unit.

Standard RNN and its variations (LSTM and GRU) are difficult to process multi-dimensional input sequences in a single model. Based on multi-dimensional RNN structure, MDLSTM is constructed to replace the single recurrent connection with many connections along all temporal properties. These connections allow the network to create a flexible internal representation of surrounding context, which is robust to handle the very long-range historical sequences.

For sequence modeling in previous studies, a one-dimensional LSTM unit consists of an input gate (*i*), forget gate (*f*), output gate (*o*), and memory cell (*c*), which control what should be stored and forgotten over long periods of time. For each LSTM unit, the gates and activations at discrete time *t* (*t* = 1, 2, …) are computed as demonstrated in Equations (8)–(13), where *x_t_*, *i_t_*, *f_t_*, *o_t_*, *c_t_*, and *h_t_* represent the unit input, input gate, forget gate, output gate, memory cell vectors, and the hidden layer output. *W* and *b* are the weighted parameter matrices and intercept parameters, *σ* denotes the logistic sigmoid function, and ⊙ denotes the Hadamard product.

(8) $$i_{t}=\sigma \left(W_{xi}\cdot x_{t}+W_{hi}\cdot h_{t-1}+\right.\left.b_{i}\right)$$

(9) $$f_{t}=\sigma \left(W_{xf}\cdot x_{t}+W_{hf}\cdot h_{t-1}+\right.\left.b_{f}\right)$$

(10) $${\tilde{c}}_{t}=\tan h\left(W_{xc}\cdot x_{t}+W_{hc}\cdot h_{t-1}\right.+\left.b_{c}\right)$$

(11) $$c_{t}={\tilde{c}}_{t}⊙i_{t}+c_{t-1}⊙f_{t}$$

(12) $$o_{t}=\sigma \left(W_{xo}\cdot x_{t}+W_{ho}\cdot h_{t-1}+\right.\left.b_{o}\right)$$

(13) $$h_{t}=o_{t}⊙\tan h\left(c_{t}\right)$$

- Attention-based Multi-Dimensional LSTM.

In the framework, 3D-LSTM units are constructed, extending a single connection to three recurrent connections for each cell’s previous state along each dimension, by using three independent forget gates. For each MDLSTM unit, the gates and activations are computed as demonstrated in Equations (14)–(21), where the inputs are arranged in 3D grids instead of a sequence, and *m*, *d*, and *r* denote the input dimensions of temporal closeness, period, and trend.

(14) $$i_{mdr}=\sigma (W^{i}⊙Z_{mdr}+W_{m}^{hi}⊙H_{\left(m-1\right)dr}+W_{d}^{hi}⊙H_{m\left(d-1\right)r}+W_{r}^{hi}⊙H_{md\left(r-1\right)}+b^{i})$$

(15) $$f_{mdr}^{m}=\sigma (W_{m}^{f}⊙Z_{mdr}+W_{m}^{hf}⊙H_{\left(m-1\right)dr}+b_{m}^{f})$$

(16) $$f_{mdr}^{d}=\sigma (W_{d}^{f}⊙Z_{mdr}+W_{d}^{hf}⊙H_{m\left(d-1\right)r}+b_{d}^{f})$$

(17) $$f_{mdr}^{r}=\sigma (W_{r}^{f}⊙Z_{mdr}+W_{r}^{hf}⊙H_{md\left(r-1\right)}+b_{r}^{f})$$

(18) $${\tilde{c}}_{mdr}=tanh(W^{c}⊙Z_{mdr}+W_{m}^{hc}⊙H_{\left(m-1\right)dr}+W_{d}^{hc}⊙H_{m\left(d-1\right)r}+W_{r}^{hc}⊙H_{md\left(r-1\right)}+b^{c})$$

(19) $$c_{mdr}={\tilde{c}}_{mdr}⊙i_{mdr}+c_{(m-1)dr}⊙f_{mdr}^{m}+c_{m\left(d-1\right)r}⊙f_{mdr}^{d}+c_{md\left(r-1\right)}⊙f_{mdr}^{r})$$

(20) $$o_{mdr}=\sigma (W^{o}⊙Z_{mdr}+W_{m}^{ho}⊙H_{\left(m-1\right)dr}+W_{d}^{ho}⊙H_{m\left(d-1\right)r}+W_{r}^{ho}⊙H_{md\left(r-1\right)}+b^{o})$$

(21) $$h_{mdr}=o_{mdr}⊙tanh(c_{mdr})$$

In 3D grids, each unit *Z_mdr_* receives three hidden vectors (*H*_(*m*-1)*dr*_, *H_m_*_(*d*-1)*r*_, *H_md_*_(*r*-1)_) and memory vectors (*c*_(*m*-1)*dr*_, *c_m_*_(*d*-1)*r*_, *c_md_*_(*r*-1)_), then computes a hidden vector *h_mdr_* and a memory vector *c_mdr_* that are passed as the next state along each of the three dimensions.

In the framework, multiple MDLSTM units can be stacked and concatenated to form more complex structures. Hence, the processing of sequence summarization and two MDLSTM layers is denoted as **MD**:

(22) $${\hat{X}}_{t+p}=MD\left(\left[Z_{t-m},\cdots ,Z_{t}\right],\left[Z_{t+1-n_{d}\cdot d},\cdots ,Z_{t+1-d}\right],\left[Z_{t+1-n_{r}\cdot r},\cdots ,Z_{t+1-r}\right],W,b\right)\;=MD\left(Z_{hist},W,b\right)$$

where $\left[Z_{t-m},\cdots ,Z_{t}\right]$, $\left[Z_{t+1-n_{d}\cdot d},\cdots ,Z_{t+1-d}\right],$ and $\left[Z_{t+1-n_{r}\cdot r},\cdots ,Z_{t+1-r}\right]$ are the recent, daily period, and weekly trends sub-sequence, which is summarized from very long-range historical input sequences. In Equation (22), *m*, *n_d_*, and *n_r_* are set to control the length of respective sub-sequence, where *d* and *r* are the number of time intervals each day and week.

### 2.5. Objective Function

During spatio-temporal features extracting in the previous section, the object is to minimize the mean squared error between the ground truths *X_t+p_* and the STNE model predictions ${\hat{X}}_{t+p}$ for model training. In this paper, the objective function of the architecture is shown in Equation (23).

(23) $$\underset{\Theta ,W,b}{\min}\sum {\left\|X_{t+p}-{\hat{X}}_{t+p}\right\|}_{2}^{2}+\alpha \|\Theta {\|}_{2}^{2}+\beta {\left\|W\right\|}_{2}^{2}$$

where the second and third term of the objective function represents the L2-norm regularization term of STNE model, which helps avoid overfitting issues, *α* and *β* refers to regularization parameters, which balance the bias-variance tradeoff.

## 3. Results

### 3.1. Dataset Description

In this study, the model is evaluated on two real-world network-scale traffic datasets, which contain key indicators and geographic information with corresponding timestamps.

The first dataset, denoted as LOOP-GS, is collected by fixed loop detectors on four connected freeways (I-5, I-405, I-90, and SR-520) in the Greater Seattle Area. This LOOP-GS dataset contains traffic variables from 323 sensor stations over the entirety of 2015 at 5 min intervals. Thus, every single node in the traffic graph contains 288 records per day.

The second dataset is from the Caltrans Performance Measurement System (PeMS), which is one of the most widely used dataset in traffic flow forecasting. The traffic variables are collected every 30 s in real-time and aggregated into 5 min intervals, from over 39,000 individual sensor stations (e.g., loops, radars) across California State. Eighty-three stations from eight freeways across Los Angeles City are selected as data sources, and the dataset is denoted as PeMS-LA. The time range covered by the PeMS-LA dataset extends from 1 May to 31 October 2017.

### 3.2. Experimental Settings

In this study, several experiments are designed to apply historical data points to forecast the traffic speed in the next 3, 6, and 12 time steps, corresponding to the future 15, 30, and 60 min. It is worth noting that these experiments focus not only on short-term (5–30 min) forecasting, but also on medium-term (30–60 min) forecasting which most prevalent approaches are not able to tackle well. To avoid using future information, chronological records of each dataset are divided into three parts: training set (70%), validation set (20%), and test set (10%).

The STNE model is compared with the following baseline models: (1) auto-regressive integrated moving average (ARIMA); (2) support vector regression (SVR); (3) feed forward neural network (FNN); (4) full-connected LSTM (FC-LSTM); and (5) graph convolutional LSTM (GC-LSTM). All experiments are implemented and tested on a Linux server (CPU: Intel(R) Xeon(R) CPU E5-2650 v4 @ 2.20 GHz, Memory: 128 GB, GPU: 2 NVIDIA GeForece GTX 1080 Ti).

In the STNE model, the Laplacian matrix *L* is calculated based on the road network characteristics and topology. For both datasets, the kernel size *K* of graph convolution is set to 4, and the number *N_f_* of kernel is 1. The average speed of all nodes is utilized as input features and set D to 1. The length of recent, periodic, and trend sub-sequence is set to 18, 4, and 4, and the dimensions of the hidden states of the MDLSTM cell are set as the amount of the nodes in the road network. The model is trained by minimizing the mean square error using RMSProp for 300 epochs with batch size 40 and initial learning rate 10^−3^.

### 3.3. Experiment Results

In this study, The performance of the proposed and baseline models are assessed by three commonly used metrics in traffic forecasting, including (1) mean absolute error (MAE) as shown in Equation (24), which evaluates differences between the prediction output and ground truth, (2) root mean square error (RMSE) as shown in Equation (25), which evaluates the absolute error, and (3) mean absolute percentage error (MAPE) as shown in Equation (26), which evaluates the relative error. Table 1 shows the comparison of STNE and baseline algorithms for 15 min, 30 min, and 60 min ahead forecasting on the two datasets, respectively.

(24) $$MAE=\frac{1}{MN}\sum_{j=1}^{M}\;\sum_{i=1}^{N}\;\left|x_{i}^{j}-{\hat{x}}_{i}^{j}\right|$$

(25) $$RMSE=\sqrt{\frac{1}{MN}\sum_{j=1}^{M}\;\sum_{i=1}^{N}\;{\left(x_{i}^{j}-{\hat{x}}_{i}^{j}\right)}^{2}}$$

(26) $$MAPE=\frac{\sum_{j=1}^{M}\;\sum_{i=1}^{N}\;\left|x_{i}^{j}-{\hat{x}}_{i}^{j}\right|}{\sum_{j=1}^{M}\;\sum_{i=1}^{N}\;\left|x_{i}^{j}\right|}$$

where $x_{i}^{j}$ and ${\hat{x}}_{i}^{j}$ denote the real and predicted traffic flow information, respectively. M is the number of samples in the time series, and N is the number of sensor stations.

The following phenomena are observed in both datasets: (1) Traditional statistical and machine learning methods may perform well for short-term forecasting, but their long-term predictions are not accurate, because the temporal dependency becomes increasingly non-linear with the growth of the horizon. (2) RNN-based methods, including FC-LSTM, GC-LSTM, and STNE, generally outperform other baselines which emphasizes the importance of modeling the temporal dependency. (3) The proposed model achieves the best performance in all evaluation metrics for all forecasting horizons, and the advantage becomes more evident with the increase of the forecasting horizon, due to its capability of modeling super-long term and graph-structured time series data.

**Figure 3:**
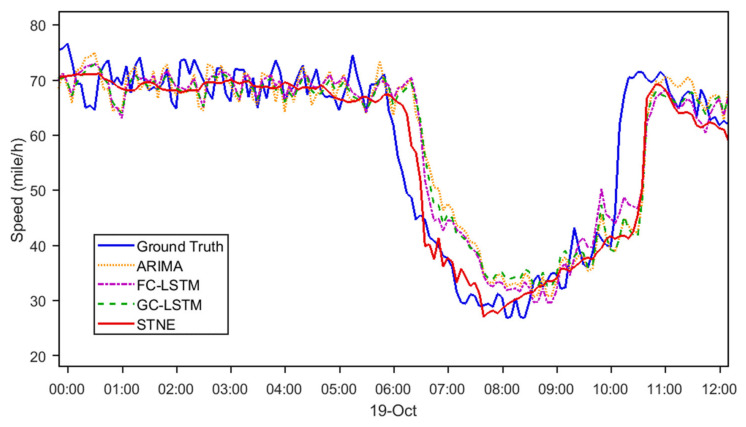
Speed forecasting for 30 min ahead in the morning peak hours (LOOP-GS).

**Figure 4:**
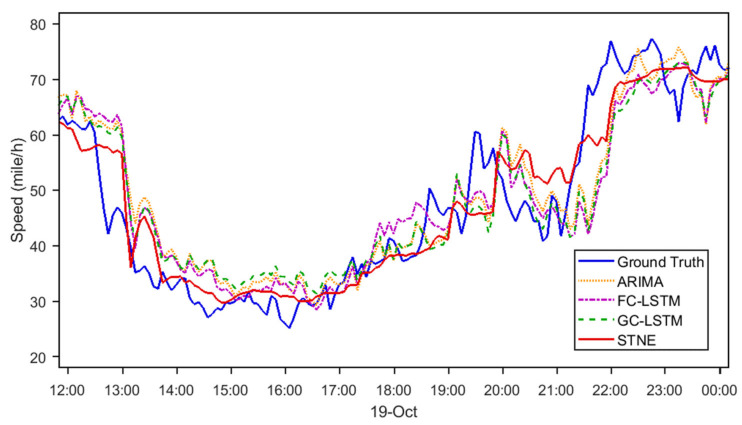
Speed forecasting for 30 min ahead in the evening peak hours (LOOP-GS).

To compare three baseline methods, ARIMA, FC-LSTM, and GC-LSTM, Figure 3 and Figure 4 show the visualization of 30 min ahead forecasting during morning and evening peak hours. The following observations are made: (1) The proposed STNE captures the trend of traffic signals more accurately even when frequent oscillation exists. This is because STNE applies network embedding technology to successfully extract periodic and trend temporal properties in the super-long time series. (2) STNE detects abrupt changes in the rush hours earlier than other baseline methods (Figure 3). Through modeling the spatial topology of the sensors, STNE is able to utilize the speed changes in neighborhood sensors for more accurate forecasting.

**Figure 5:**
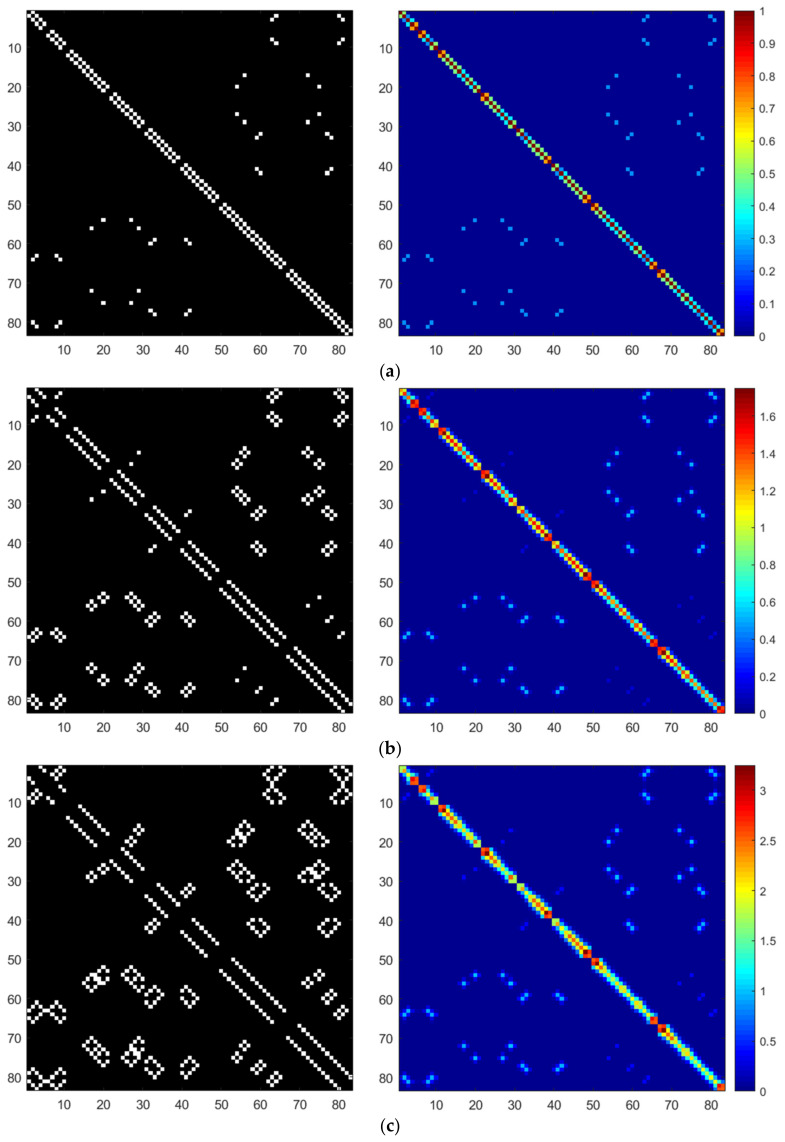

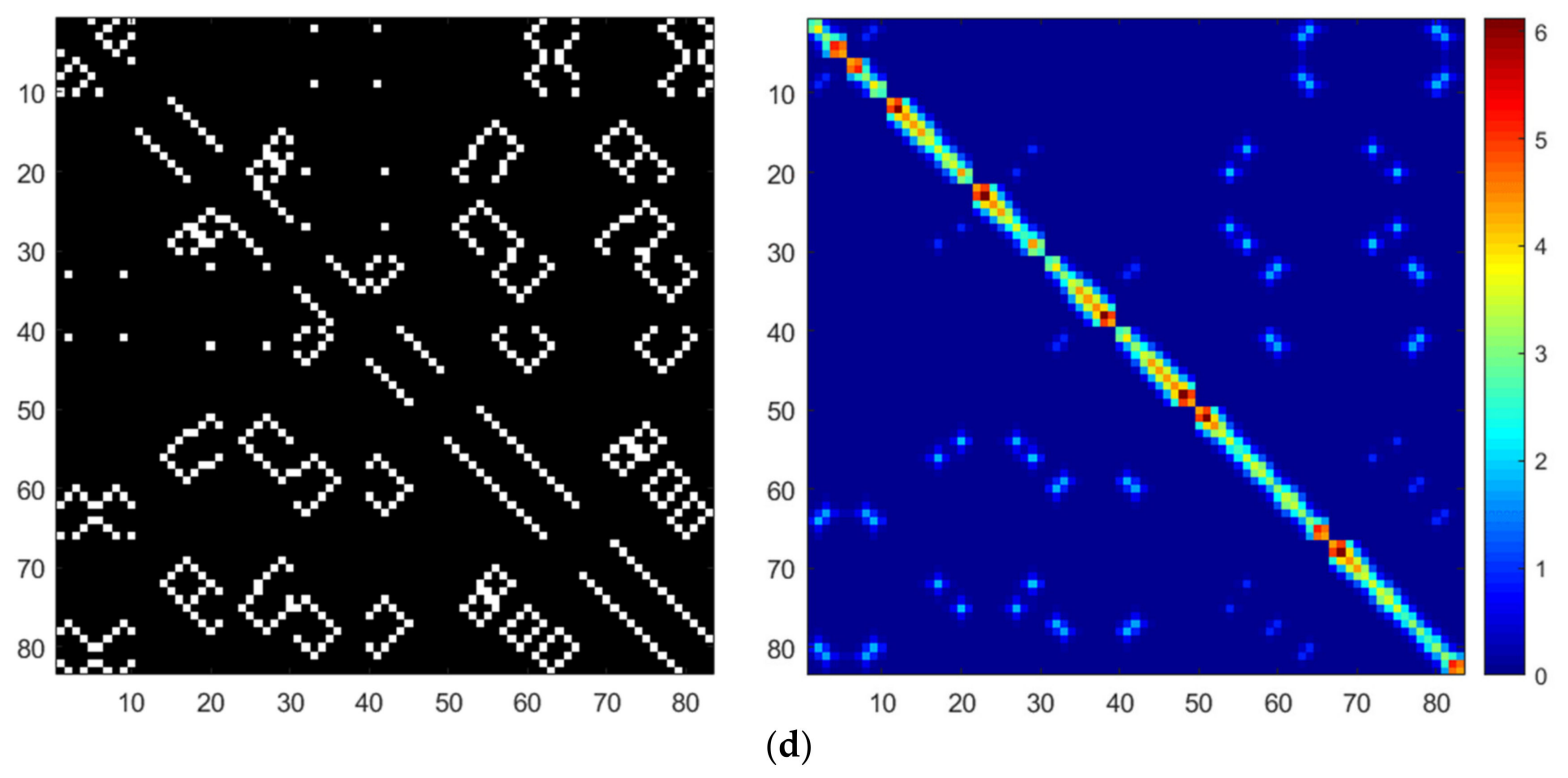
Nearest neighbor and correlation coefficient. (**a**) First neighbor and correlation coefficient. (**b**) Second neighbor and correlation coefficient. (**c**) Third neighbor and correlation coefficient. (**d**) Fourth neighbor and correlation coefficient.

Figure 5 presents the spatial correlation between the target node and its nearest neighbors in the PeMS-LA dataset. The left panels of Subfigures (a) through (d) depict the positions of the first, second, third, and fourth nearest neighbors for each point, represented in white according to the hop count between nodes. The right panels of Subfigures (a) through (d) illustrate the corresponding variations in spatial correlation as additional neighbors are incorporated into the spatial model. The results indicate that as the hop count between the target node and its neighbors increases, the correlation generally decreases. Notably, the hop count does not necessarily correspond to the physical distance between nodes. This analysis improves the interpretability of the model and is critical for informed selection of model parameters.

Table 2 shows the effects of different parameters for 30 min ahead forecasting on the LOOP-GS dataset. *K* corresponds to the kernel size of the graph convolution filter, and (*m*, *n_d_*, *n_r_*) corresponds to the sub-sequence length of temporal closeness, period, and trend. Larger *K* and each temporal sub-sequence sizes enable the model to capture broader spatial-temporal dependency at the cost of increasing learning complexity. With an increase in most parameters, the error (e.g., MAPE) on the LOOP-GS dataset initially decreases and then increases, as additional information introduces distractions.

## 4. Discussion

In this study, the performance of the spatio-temporal network embedding (STNE) model for traffic forecasting was evaluated using two extensive datasets, LOOP-GS and PeMS-LA. The analysis indicates that STNE significantly outperforms traditional statistical and machine learning methods in both short-term and medium-term traffic forecasting, underscoring its effectiveness in managing complex spatio-temporal dependencies.

### 4.1. Performance Comparison

The results demonstrate that traditional methods, such as ARIMA and SVR, while competent for short-term forecasting (15 min), encounter difficulties with longer time horizons (30 and 60 min). This limitation is attributed to the increasing non-linearity of temporal dependencies as the forecasting horizon extends. These methods do not capture complex patterns and trends over longer periods, highlighting the necessity for more advanced models capable of addressing such non-linearities.

In contrast, RNN-based methods, including FC-LSTM, GC-LSTM, and STNE, exhibit superior performance across all forecasting horizons. This improvement is particularly evident in STNE, which consistently surpasses the baseline methods in all evaluation metrics (MAE, RMSE, MAPE) for both datasets. The advantage of STNE becomes more pronounced with longer forecasting horizons, attributable to its enhanced capacity to model long-term dependencies and its integration of graph-structured time series data.

### 4.2. Insights from Visualization

The visualizations of 30 min ahead forecasting during peak hours (Figure 3 and Figure 4) provide further insights into the strengths of STNE. The model effectively captures traffic trends and handles oscillations better than baseline methods. The ability of STNE to accurately model periodic and trend temporal properties proves crucial for forecasting traffic during dynamic conditions, such as rush hours. Additionally, STNE’s capability to detect abrupt changes in traffic patterns earlier than other models highlights its advantage in timely and accurate forecasting.

The success of STNE in capturing trends and detecting changes is attributed to its use of network embedding technology. This approach enables STNE to leverage spatial topology and the interactions between neighboring sensors, resulting in more precise predictions. The incorporation of spatial dependencies enhances the model’s performance, particularly within complex traffic networks.

### 4.3. Impact of Hyperparameters

The analysis of different hyperparameters (Table 2) reveals the impact of various parameters on forecasting accuracy. The kernel size (*K*) and the lengths of temporal sub-sequences (*m*, *n_d_*, *n_r_*) play a critical role in shaping the model’s performance. While larger values for these parameters allow the model to capture broader spatio-temporal dependencies, they also increase computational complexity and can lead to overfitting if not managed properly. The observed pattern, where MAPE decreases and then increases with parameter values, suggests that an optimal range for each hyperparameter exists, balancing model complexity and forecasting accuracy.

## 5. Conclusions

In summary, STNE represents a significant advancement in traffic forecasting, demonstrating superior performance over traditional and baseline methods. The ability of STNE to model complex spatio-temporal dependencies and accurately forecast traffic across different horizons positions it as a valuable tool for traffic management and planning.

### 5.1. Potential Application

The STNE model’s flexibility extends beyond traffic forecasting, making it a versatile tool for other domains involving graph-structured time series data. The model’s ability to effectively manage complex spatial and temporal dependencies over extended input sequences allows for its application in both short-term and long-term forecasting scenarios across different fields:

- Weather Forecasting.
  - Short-term application: The STNE model can be adapted for short-term weather forecasting by analyzing sensor data from weather stations. By focusing on fine-grained temporal patterns such as hourly fluctuations in temperature, humidity, and precipitation, the model enhances the accuracy of short-term weather predictions by effectively capturing sudden weather changes.
  - Long-term application: For long-term weather forecasting, the STNE model can be applied to analyze seasonal patterns and long-term climate trends. Extensive historical weather data can be processed to predict future conditions over extended periods, providing valuable insights into potential climate change impacts.
- Car-Hailing Supply–Demand Analysis.
  - Short-term application: The STNE model can be utilized to predict real-time demand for car-hailing services in various urban areas, leveraging historical booking data and current traffic conditions. Localized patterns such as peak hours and demand surges are focused on, allowing for the optimization of fleet management and improvement of service availability in real-time.
  - Long-term application: In long-term supply–demand analysis, the STNE model can be used to forecast trends in car-hailing demand over different seasons or years. This capability aids in strategic resource allocation and long-term fleet management, enabling service providers to optimize operations over extended periods.

### 5.2. Limitations

Despite its notable performance, the STNE model has several limitations. The model’s dependence on precise spatial network representations means that inaccuracies in the graph topology can potentially impact prediction accuracy. Additionally, although STNE performs well in capturing long-term dependencies, its effectiveness in real-time traffic management and adaptability to varying traffic conditions and network structures remains an area for further investigation.

The influence of random factors, such as accidents or roadwork, also poses a challenge. These unpredictable events can significantly alter traffic patterns and introduce variability that may not be fully accounted for by the model. Addressing these random influences is crucial, as they can markedly affect forecast accuracy.

### 5.3. Applicability for Traffic Management

The STNE model offers several opportunities for enhancing the quality of traffic management through its advanced forecasting capabilities:

- Optimized Traffic Control.
  - Dynamic traffic signal control: Enhanced traffic pattern predictions enable more effective dynamic traffic signal control. By anticipating traffic flow and congestion, traffic signals can be adjusted in real-time to reduce delays and improve traffic flow.
  - Adaptive traffic management systems: The STNE model facilitates the development of adaptive traffic management systems capable of responding dynamically to traffic condition changes, thereby optimizing traffic management strategies and mitigating congestion.
- Enhanced Route Planning.
  - Real-time route optimization: Accurate traffic forecasts improve real-time route planning, assisting drivers in avoiding congested areas and reducing travel time. This capability benefits navigation systems and GPS services.
  - Predictive Travel Time Estimates: Improved traffic condition forecasts offer more reliable travel time estimates, aiding drivers in planning trips and making informed decisions regarding departure times.
- Efficient Public Transportation.
  - Transit scheduling: Accurate predictions of traffic conditions and passenger demand enhance the scheduling of public transportation services, leading to reduced wait times, increased service frequency, and optimized resource allocation.
  - Demand-responsive transit: The STNE model supports the development of demand-responsive transit systems that adjust routes and schedules based on real-time and forecasted demand, thereby improving public transportation efficiency and effectiveness.

### 5.4. Future Work

Future research should focus on refining the model’s hyperparameters, exploring alternative network embeddings, and incorporating additional data sources, such as weather conditions or special events, to improve forecasting accuracy. Moreover, extending the model to handle multi-modal traffic data and assessing its performance in diverse geographical contexts could provide valuable insights into its robustness and applicability.

## Figures and Tables

**Table 1:** Performance comparison of different approaches for all forecasting horizons on the LOOP-GS and PeMS-LA datasets.

Model	LOOP-GS			PeMS-LA		
	MAE	RMSE	MAPE	MAE	RMSE	MAPE
15 min						
ARIMA	3.19	5.26	8.72%	3.03	5.24	7.87%
SVR	3.09	5.27	8.01%	2.96	5.28	7.52%
FNN	3.08	5.05	8.32%	2.96	5.11	7.81%
LSTM	3.03	5.04	7.85%	2.85	5.06	7.36%
GC-LSTM	2.88	4.64	7.24%	2.84	4.84	7.26%
STNE	**2.23**	**3.72**	**5.49%**	**2.01**	**3.70**	**5.14%**
30 min						
ARIMA	3.89	6.58	11.71%	4.59	7.70	12.57%
SVR	3.69	6.60	10.32%	4.31	7.78	11.28%
FNN	3.73	6.24	10.97%	4.33	7.35	11.97%
LSTM	3.65	6.24	9.93%	4.16	7.36	10.91%
GC-LSTM	3.43	5.75	9.05%	4.14	7.02	10.87%
STNE	**2.91**	**4.90**	**7.44%**	**3.20**	**5.63**	**8.33%**
60 min						
ARIMA	5.07	8.44	16.72%	6.85	10.55	19.83%
SVR	4.72	8.45	14.86%	6.18	10.74	17.00%
FNN	4.79	7.87	15.44%	6.23	9.91	18.03%
LSTM	4.71	7.88	13.82%	5.85	9.75	15.90%
GC-LSTM	4.29	7.21	11.88%	5.50	9.11	14.93%
STNE	**3.88**	**6.64**	**10.68%**	**4.46**	**7.60**	**11.83%**

**Table 2:** Effects of kernel size K of graph convolution and the sequence size of temporal closeness, period, and trend.

(*m*, *n_d_*, *n_r_*)	MAPE (*K* = 3)	MAPE (*K* = 4)	MAPE (*K* = 5)
(12, 3, 3)	8.12%	7.91%	7.85%
(18, 3, 3)	7.93%	7.74%	7.76%
(24, 3, 3)	8.04%	7.85%	7.78%
(18, 4, 4)	7.82%	7.57%	7.62%
(18, 6, 4)	7.79%	7.67%	7.61%
(12, 3, 3)	8.12%	7.91%	7.85%

## Data Availability

The first dataset, denoted as LOOP-GS, is collected by fixed loop detectors on four connected freeways (I-5, I-405, I-90, and SR-520) in the Greater Seattle Area, and can be downloaded from this website: https://github.com/zhiyongc/Seattle-Loop-Data, accessed on 26 December 2019. The second dataset Caltrans Performance Measurement System (PeMS) can be downloaded from this website: https://pems.dot.ca.gov/, accessed on 10 August 2020.

## References

[1] Lana I., Del Ser J., Velez M., Vlahogianni E.I. (2018). Road traffic forecasting: Recent advances and new challenges. IEEE Intell. Transp. Syst. Mag..

[2] Williams B.M., Hoel L.A. (2003). Modeling and forecasting vehicular traffic flow as a seasonal ARIMA process: Theoretical basis and empirical results. J. Transp. Eng..

[3] Thomas T., Weijermars W., Van Berkum E. (2010). Predictions of urban volumes in single time series. IEEE Trans. Intell. Transp. Syst..

[4] Guo J., Williams B.M. (2010). Real-Time Short-Term Traffic Speed Level Forecasting and Uncertainty Quantification Using Layered Kalman Filters. Transp. Res. Rec. J. Transp. Res. Board.

[5] Ghosh B., Basu B., O’Mahony M. (2009). Multivariate short-term traffic flow forecasting using time-series analysis. IEEE Trans. Intell. Transp. Syst..

[6] Castro-Neto M., Jeong Y.-S., Jeong M.-K., Han L.D. (2009). Online-SVR for short-term traffic flow prediction under typical and atypical traffic conditions. Expert Syst. Appl..

[7] Castillo E., Nogal M., Menendez J.M., Sanchez-Cambronero S., Jimenez P. (2012). Stochastic demand dynamic traffic models using generalized beta-Gaussian Bayesian networks. IEEE Trans. Intell. Transp. Syst..

[8] Atluri G., Karpatne A., Kumar V. (2018). Spatio-temporal data mining: A survey of problems and methods. ACM Comput. Surv. (CSUR).

[9] Huang W., Song G., Hong H., Xie K. (2014). Deep architecture for traffic flow prediction: Deep belief networks with multitask learning. IEEE Trans. Intell. Transp. Syst..

[10] Lv Y., Duan Y., Kang W., Li Z., Wang F.-Y. (2015). Traffic flow prediction with big data: A deep learning approach. IEEE Trans. Intell. Transp. Syst..

[11] Yang H.F., Dillon T.S., Chen Y.P.P. (2017). Optimized structure of the traffic flow forecasting model with a deep learning approach. IEEE Trans. Neural Netw. Learn. Syst..

[12] Ma X., Tao Z., Wang Y., Yu H., Wang Y. (2015). Long short-term memory neural network for traffic speed prediction using remote microwave sensor data. Transp. Res. Part C Emerg. Technol..

[13] Tian Y., Pan L. Predicting short-term traffic flow by long short-term memory recurrent neural network. Proceedings of the IEEE International Conference on Smart City Socialcom Sustaincom.

[14] Yu R., Li Y., Shahabi C., Demiryurek U., Liu Y. Deep learning: A generic approach for extreme condition traffic forecasting. Proceedings of the 2017 SIAM International Conference on Data Mining.

[15] Ke J., Zheng H., Yang H., Chen X. (2017). Short-term forecasting of passenger demand under on-demand ride services: A spatio-temporal deep learning approach. Transp. Res. Part C Emerg. Technol..

[16] Wu Y., Tan H. (2016). Short-term traffic flow forecasting with spatial-temporal correlation in a hybrid deep learning framework. arXiv.

[17] Ma X., Dai Z., He Z., Ma J., Wang Y., Wang Y. (2017). Learning traffic as images: A deep convolutional neural network for large-scale transportation network speed prediction. Sensors.

[18] Zhang J., Zheng Y., Qi D. Deep spatio-temporal residual networks for citywide crowd flows prediction. Proceedings of the Thirty-First AAAI Conference on Artificial Intelligence.

[19] Zhang J., Zheng Y., Qi D., Li R., Yi X. DNN-based prediction model for spatio-temporal data. Proceedings of the 24th ACM SIGSPATIAL International Conference on Advances in Geographic Information Systems.

[20] Yu H., Wu Z., Wang S., Wang Y., Ma X. (2017). Spatiotemporal recurrent convolutional networks for traffic prediction in transportation networks. Sensors.

[21] Yao H., Wu F., Ke J., Tang X., Jia Y., Lu S., Gong P., Ye J., Li Z. Deep multi-view spatial-temporal network for taxi demand prediction. Proceedings of the Thirty-Second AAAI Conference on Artificial Intelligence.

[22] Yao H., Liu Y., Wei Y., Tang X., Li Z. (2019). Learning from Multiple Cities: A Meta-Learning Approach for Spatial-Temporal Prediction. arXiv.

[23] Yu B., Yin H., Zhu Z. (2017). Spatio-temporal graph convolutional networks: A deep learning framework for traffic forecasting. arXiv.

[24] Li Y., Yu R., Shahabi C., Liu Y. (2017). Diffusion convolutional recurrent neural network: Data-driven traffic forecasting. arXiv.

[25] Geng X., Li Y., Wang L., Zhang L., Yang Q., Ye J., Liu Y. (2019). Spatiotemporal multi-graph convolution network for ride-hailing demand forecasting. Proc. AAAI Conf. Artif. Intell..

[26] Yao H., Tang X., Wei H., Zheng G., Li Z. (2019). Revisiting spatial-temporal similarity: A deep learning framework for traffic prediction. Proc. AAAI Conf. Artif. Intell..

[27] Ma C., Dai G., Zhou J. (2021). Short-term traffic flow prediction for urban road sections based on time series analysis and LSTM_BILSTM method. IEEE Trans. Intell. Transp. Syst..

[28] Wang K., Ma C., Qiao Y., Lu X., Hao W., Dong S. (2021). A hybrid deep learning model with 1DCNN-LSTM-Attention networks for short-term traffic flow prediction. Phys. A Stat. Mech. Its Appl..

[29] Shu W., Cai K., Xiong N.N. (2021). A short-term traffic flow prediction model based on an improved gate recurrent unit neural network. IEEE Trans. Intell. Transp. Syst..

[30] Berlotti M., Di Grande S., Cavalieri S. (2024). Proposal of a machine learning approach for traffic flow prediction. Sensors.

[31] Rossi D., Pascale A., Mascolo A., Guarnaccia C. (2024). Coupling different road traffic noise models with a multilinear regressive model: A measurements-independent technique for urban road traffic noise prediction. Sensors.

[32] Huang X., Wang J., Lan Y., Jiang C., Yuan X. (2023). MD-GCN: A multi-scale temporal dual graph convolution network for traffic flow prediction. Sensors.

[33] Ji J., Wang J., Huang C., Wu J., Xu B., Wu Z., Zhang J., Zheng Y. (2023). Spatio-temporal self-supervised learning for traffic flow prediction. Proc. AAAI Conf. Artif. Intell..

[34] Li M., Li M., Liu B., Liu J., Liu Z., Luo D. (2022). Spatio-temporal traffic flow prediction based on coordinated attention. Sustainability.

[35] Cui Z., Zhang J., Noh G., Park H.J. (2022). Mfdgcn: Multi-stage spatio-temporal fusion diffusion graph convolutional network for traffic prediction. Appl. Sci..

[36] Li W., Zhan X., Liu X., Zhang L., Pan Y., Pan Z. (2023). SASTGCN: A Self-Adaptive Spatio-Temporal Graph Convolutional Network for Traffic Prediction. ISPRS Int. J. Geo-Inf..

[37] Ma H., Qin X., Jia Y., Zhou J. (2023). Dynamic Spatio-Temporal Graph Fusion Convolutional Network for Urban Traffic Prediction. Appl. Sci..

